# Perceived Neighborhood Environment Associated with Sarcopenia in Urban-Dwelling Older Adults: The Korean Frailty and Aging Cohort Study (KFACS)

**DOI:** 10.3390/ijerph18126292

**Published:** 2021-06-10

**Authors:** Yuri Seo, Miji Kim, Hyungeun Shin, Changwon Won

**Affiliations:** 1 Department of Biomedical Science and Technology, Graduate School, Kyung Hee University, Seoul 02447, Korea; dkddkkd55@naver.com (Y.S.); she9310@hanmail.net (H.S.); 2 Department of Biomedical Science and Technology, East-West Medical Research Institute, College of Medicine, Kyung Hee University, Seoul 02447, Korea; 3 Elderly Frailty Research Center, Department of Family Medicine, College of Medicine, Kyung Hee University, Seoul 02447, Korea

**Keywords:** environment, sarcopenia, cohort study, aging

## Abstract

Sarcopenia is associated with adverse health outcomes among older individuals. However, little is known about its association with neighborhood environmental factors. We explored the relationship between sarcopenia and perceived neighborhood environmental factors among community-dwelling older adults aged 70–84 years. We analyzed 1778 participants (mean age of 75.9 ± 3.8 years; 54.0% women) who lived in urban areas and underwent dual-energy X-ray absorptiometry from the Korean Frailty and Aging Cohort Study. Sarcopenia was defined according to the Asian Working Group for Sarcopenia 2019 definition. Perceived neighborhood environmental factors were assessed using the Environmental Module of the International Physical Activity Questionnaire (IPAQ-E). In the multivariate analysis, compared to the fifth quintile of the IPAQ-E score, the odds ratios (ORs) and 95% confidence intervals (CIs) for sarcopenia in the first, second, third, and fourth quintiles were 2.13 (1.40–3.24), 1.72 (1.12–2.64), 1.75 (1.15–2.66), and 1.62 (1.06–2.47), respectively. These neighborhood environmental characteristics were linked with an increased likelihood of sarcopenia: no public transportation access (OR = 2.04; 95% CI = 1.19–3.48), poor recreational facilities access (OR = 1.39; 95% CI = 1.01–1.90), absence of destination (OR = 1.53; 95% CI = 1.06–2.20), many hill hazards (OR = 1.36; 95% CI = 1.03–1.78), and lack of traffic safety (OR = 1.35; 95% CI = 1.02–1.78). Thus, better neighborhood environmental strategies may help prevent sarcopenia among urban-dwelling older adults.

## 1. Introduction

Sarcopenia is related to adverse health outcomes such as falls, functional decline, frailty, and mortality [1], which cause personal, social, and economic burden among older adults [2,3]. The prevalence of sarcopenia is around 10% in community-dwelling older adults aged ≥60 years, and its incidence may increase with every one-year increase in age [4]. Therefore, it is important to identify the risk factors that affect the development of sarcopenia. Risk factors for sarcopenia include physical inactivity, poor nutritional status, inflammation, oxidative stress, and chronic diseases [1,5]. Most of these risk factors can be prevented through lifestyle changes; however, environmental support is needed to maintain or achieve a healthy lifestyle [6]. 

The World Health Organization (WHO) emphasized the role of the physical and social neighborhood environment for “healthy and active aging” in older adults in its guidelines of global age-friendly cities [7,8]. Many studies have previously reported that a poor neighborhood environment is associated with adverse health outcomes, including falls [9], disability [10], frailty [11,12], and mortality [13] all of which are known to be related to sarcopenia. Previous studies have reported that the neighborhood environment promotes healthy lifestyle habits, such as increased physical and social activity [13,14,15] and better dietary choices in older adults [16,17]. Therefore, the neighborhood environment may have implications in the prevention of sarcopenia because it provides older adults opportunities to participate in physical and social activities.

A longitudinal study found that neighborhood environmental factors that were objectively measured using geographic information systems were related to declines in muscle mass and grip strength in older adults [18]. Another study confirmed the association between neighborhood walkability using the Walk Score website and risk of sarcopenia [16]. However, these studies did not analyze various neighborhood environmental factors, including the walking environment, aesthetics, and safety, etc. In addition, we previously identified the relationship between physical frailty and the perceived neighborhood environment [12]. As a result of the study, physical frailty was related to the IPAQ-E total score, access to destinations, and neighborhood safety factors [12]. Sarcopenia shares common risk factors with physical frailty [5], it may be related to various neighborhood environmental factors. Therefore, using a structured questionnaire, we aimed to investigate the relationship between perceived neighborhood environment and sarcopenia, as diagnosed using the Asian Working Group for Sarcopenia (AWGS) criteria among urban-dwelling older adults.

## 2. Materials and Methods

### 2.1. Study Design and Participants

**Figure 1:**
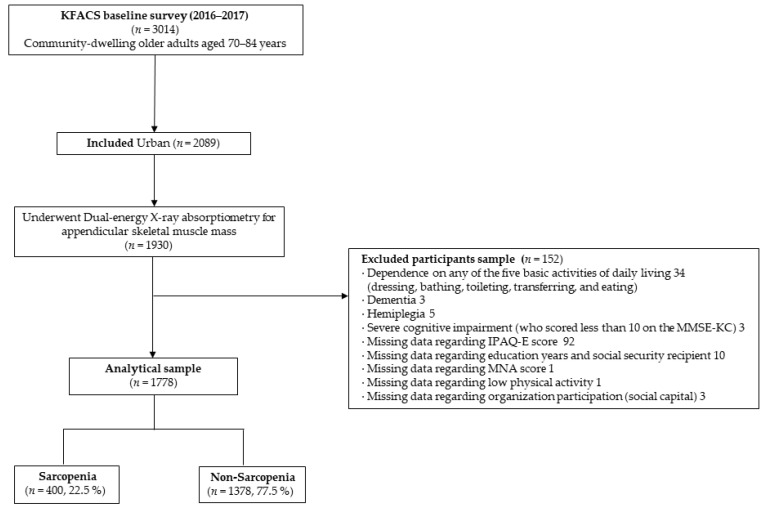
Flow chart of the study population.

This study analyzed baseline data obtained from the Korean Frailty and Aging Cohort Study (KFACS). The KFACS is a nationwide, longitudinal cohort study of older adults aged 70–84 years, and the baseline survey was conducted from 2016 to 2017. The participants were recruited from age- and sex-stratified community residents of 10 centers in rural, sub-urban, and urban areas; the ratio of the age groups 70–74 years, 75–79 years, and 80–84 years was 6:5:4, and the male:female sex ratio was 1:1 [19]. A total of 3014 individuals participated in the baseline KFACS, and participants who were living in rural areas (*n* = 909) and had missing data regarding residence (*n* = 16) were excluded. Consequently, only participants who were living in urban areas and underwent dual-energy X-ray absorptiometry (DXA) for appendicular skeletal muscle mass were included (*n* = 1930). The exclusion criteria were as follows: (i) dependence on any of the five basic activities of daily living (ADL; *n* = 34); (ii) clinical diagnosis of dementia (*n* = 3); (iii) history of hemiplegia (*n* = 5); and (iv) severe cognitive impairment, which was defined as a score of <10 in the Mini-Mental State Examination (MMSE; *n* = 3). A total of 107 participants were excluded for having missing data regarding the IPAQ-E scores (*n* = 92), education years and social security recipient (*n* = 10), Mini Nutritional Assessment (MNA) scores (*n* = 1), low physical activity (*n* = 1), and organizational participation (*n* = 3). The final sample comprised 1778 participants (Figure 1). This study received ethical approval for the KFACS protocol from the Clinical Research Ethics Committee of Kyung Hee University (Institutional Review Board [IRB] number: 2015-12-103). The present study was exempt from requiring approval by the IRB of the Clinical Research Ethics Committee of Kyung Hee University Medical Center (IRB number: 2021-01-032).

### 2.2. Perceived Neighborhood Environment Assessment

Perceived neighborhood environment was assessed using the IPAQ-E (self-report format) [20]. The IPAQ-E is a tool developed to evaluate perceived environmental factors that affect physical activity and has been used in many countries [21,22,23]. The reliability of the questionnaire has been verified [24]. Neighborhood environment in the IPAQ-E is defined as a 10–15-min walk from the home, which includes environmental characteristics consisting of 17 items regarding both physical and social environmental factors, and the items are categorized into seven components [20]: (i) residential density (one item: type of housing); (ii) access to destinations (five items: perceived range or accessibility to the destination by walk, which include shops, public transports, recreational facilities, destination [bank, hospital], and hill hazards); (iii) neighborhood infrastructure (four items: perceived safety for walking and cycling, which include presence and maintenance of sidewalks and bike lanes); (iv) neighborhood safety (four items: perceived safety for crime and traffic, which include crime safety at night and during the day, traffic safety, and traffic safety for bicyclists); (v) social environment (one item: seeing people being active); (vi) aesthetic qualities (one item: perceived attractiveness of landscape and pleasantness of places); and (vii) street connectivity (one item: connectivity of streets) [20]. Appendix A provides the 17 items and the methods for scoring them. Each item has a possible score from 1 to 4, except for “type of housing,” which is scored from 0 to 1; the total possible score is from 16 to 65, with higher scores indicating better perceived neighborhood environment [12,25]. In addition, the dichotomized variable is coded as “0” for 1–2 points and “1” for 3–4 points [12].

### 2.3. Definition of Sarcopenia

Sarcopenia was defined as low muscle mass (appendicular skeletal muscle mass [ASM] index of <7.0 kg/m^2^ in men and <5.4 kg/m^2^ in women) combined with low muscle strength (hand grip strength [HGS] of <28 kg in men and <18 kg in women) or low physical performance (defined for both men and women as a low result in at least one of the three physical performance measures: usual gait speed of <1.0 m/s; sit-to-stand test score of ≥12 s; or low Short Physical Performance Battery [SPPB] score of ≤9), according to the criteria established by the AWGS 2019 consensus [26]. Three components for evaluating sarcopenia were measured as follows: (i) ASM was assessed using DXA (Hologic DXA, Hologic Inc., Bedford, MA, USA; Lunar, GE Healthcare, Madison, WI, USA) [27], which measures the sum of the lean mass of the right and left arms and legs; (ii) HGS was measured twice in each hand while in a standing position, using a digital hand grip dynamometer (T.K.K.5401; Takei Scientific Instruments Co, Ltd, Tokyo, Japan), and the highest value was used for the analysis [28]; and (iii) low physical performance was assessed using the gait speed, sit-to-stand test score, and SPPB score. The gait speed was assessed over 4 m using an automatic gait speed meter (Dynamic Physiology, Daejeon, Korea), with acceleration and deceleration phases of 1.5 m each. The test was repeated twice, and the average of the two trials was used for the analysis. The sit-to-stand test measured the time it took to get up from and sit on a chair for a total of five times as quickly as possible, with arms across their chest. The time required to finish five repetitions was used for the analysis [29]. The SPPB independently investigated three items: balance test (side-by-side stance, semi-tandem stance, and tandem stance), usual gait speed, and five-times sit-to-stand test. Each item of the SPPB has a possible score of 0–4, with the total possible score ranging from 0 to 12 [30].

### 2.4. Other Measurements

#### 2.4.1. Sociodemographic Variables 

Demographic characteristics included residence, age, sex, years of education, living conditions, marital status, and social security recipient. ADL was assessed using the Korean Activities of Daily Living (KADL) scale [31]. 

#### 2.4.2. Health-Related and Lifestyle Variables 

Health-related and lifestyle factors included body mass index (BMI), medical history, smoking status, and alcohol consumption (≥2–3 times/week). Low physical activity was defined as <494.65 kcal for men and <283.50 kcal for women (as assessed by the International Physical Activity Questionnaire), with these values corresponding to the lowest 20% of the total consumed energy established among the general population of Korean older adults [19]. Nutritional status was evaluated using the Korean version of the short-form Mini Nutritional Assessment (MNA-SF) [32]. The following comorbidities were recorded: those related to the circulatory system (hypertension, dyslipidemia, myocardial infarction, congestive heart failure, angina pectoris, peripheral vascular disease, and cerebrovascular disease), those related to the musculoskeletal system and connective tissue (osteoarthritis, rheumatoid arthritis, and osteoporosis), and those related to the respiratory system (asthma and chronic obstructive pulmonary disease), according to doctors’ diagnoses as self-reported by the participants. 

#### 2.4.3. Psychosocial or Social Variables 

Psychosocial and social variables, including self-perceived health, were assessed using the EuroQoL five-dimension (EQ-5D) scale [33], and the answers were classified as good or poor. Depressive symptoms were evaluated using the Korean version of the Short Form Geriatric Depression Scale (SGDS-K) [34]. Cognitive function was determined using the Mini-Mental State Examination (MMSE) [35]. Participation in social meetings was classified as yes or no.

### 2.5. Statistical Analysis

Descriptive statistics were used to analyze the participants’ characteristics. Independent *t*-tests and chi-square tests were used to describe the differences between the non-sarcopenia and sarcopenia groups. The characterization comparisons of the sarcopenia group were analyzed using univariate logistic regression and described as odds ratios (ORs) using 95% confidence intervals (CIs). Multivariate logistic regression analyses were used to ascertain the association between sarcopenia and neighborhood environmental factors in urban areas. The following covariates were included in the logistic regression models: Model 1, adjusted for sociodemographic variables (age, sex, education, living alone, marital status, and social security recipient) and DXA equipment used; Model 2, further adjusted for health-related and lifestyle variables (smoking status, alcohol consumption, number of comorbidities, nutritional status, low physical activity, and BMI); Model 3, further adjusted for psychosocial variables (depressive symptoms and cognitive function); and Model 4, further adjusted for social variables (participation in social meetings). Moreover, we used logistic regression to evaluate the association between sarcopenia and the IPAQ-E score quintiles, controlling for sociodemographic variables and DXA equipment used, health-related and lifestyle variables, psychosocial variables, and social variables. We used the relative risk (RR) for sarcopenia related to neighborhood environmental risk factors. In addition, estimates of the attributable risk (AR) and population AR (PAR) were calculated to identify the most significant neighborhood environmental factors contributing to sarcopenia. All the statistical analyses were performed using the SPSS software (ver. 25.0; IBM Corp., Armonk, NY, USA), with statistical significance set at *p* < 0.05.

## 3. Results

### 3.1. Characteristics of the Study Population

The characteristics of participants with and without sarcopenia are shown in Table 1. Of the 1778 older adults (54.0% women, mean age of 75.9 years), 400 (22.5%) had sarcopenia. Participants with sarcopenia were more likely to be older (*p* < 0.001) and women (*p* = 0.006). Regarding the health-related factors, the sarcopenia group had a higher smoking rate, higher prevalence of diabetes and osteoporosis (*p* < 0.05). Moreover, the sarcopenia group had lower physical activity levels, poorer nutritional status, a lower BMI, and took more medications (all *p* < 0.001). Compared with those without sarcopenia, those with sarcopenia were more likely to perceive their health as poorer (*p* < 0.001), have more depressive symptoms (*p* < 0.001), and have worse cognitive function (*p* = 0.001). Additionally, those with sarcopenia seemed to have lower social participation levels (*p* < 0.05). There was a significant difference between the sarcopenia and non-sarcopenia groups regarding the HGS, gait speed, five-times sit-to-stand test score, SPPB score, and skeletal muscle mass index (all *p* < 0.001). 

### 3.2. Neighborhood Environmental Factors Associated with Sarcopenia

The non-sarcopenia group had a significantly higher IPAQ-E score (*p* = 0.005), access to destinations score (*p* < 0.001), and neighborhood safety score (*p* = 0.035) (Table 1). Table 2 shows the association between neighborhood environmental factors and sarcopenia. Logistic regression analyses showed that no access to public transport (OR = 2.04; 95% CI = 1.19–3.48), poor access to recreational facilities (OR = 1.39; 95% CI = 1.01–1.90), absence of destination (OR = 1.53; 95% CI = 1.06–2.20), many hill hazards (OR = 1.36; 95% CI = 1.03−1.78), and lack of traffic safety (OR = 1.35, 95% CI = 1.02−1.78) were significantly associated with sarcopenia after adjusting for potentially confounding variables (Model 4). 

We further identified the AR and population PAR of five perceived neighborhood environmental factors with significance in the logistic regression analysis for sarcopenia, as presented in Appendix A, Table A2. Among the five environmental factors, no access to public transportation was the most significant contributing factor for sarcopenia (Appendix A, Table A2).

### 3.3. Neighborhood Environmental Total Score Associated with Sarcopenia

**Figure 2:**
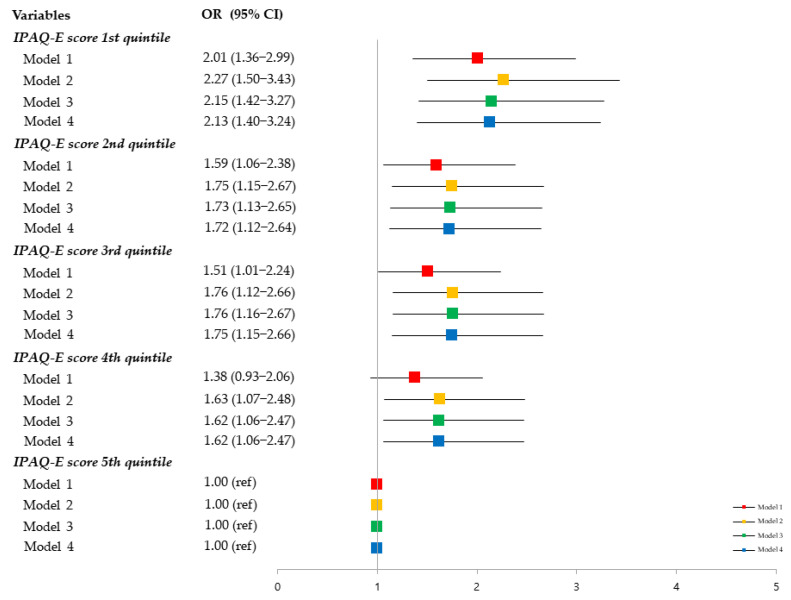
Adjusted odds ratios (ORs) and 95% confidence intervals (CIs) of the IPAQ-E score quintiles for sarcopenia. Model 1: Adjusted for sociodemographic variables (age, sex, education, living alone, marital status, and social security recipient) and DXA equipment used. Model 2: Further adjusted for health-related lifestyle variables (smoking status, alcohol consumption, number of comorbidities, nutritional status, low physical activity, and body mass index). Model 3: Further adjust for psychosocial variables (depressive symptoms and cognitive function). Model 4: Further adjusted for social variables (participation in social meetings).

Figure 2 shows the adjusted odds ratios of the IPAQ-E total score quintiles for sarcopenia. After adjusted for potentially confounding variables (Model 4), compared to the IPAQ-E total score in the fifth quintile, the ORs for sarcopenia in the first, second, third, and fourth quintiles were 2.13 (95% CI = 1.40–3.24), 1.72 (95% CI = 1.12–2.64), 1.75 (95% CI = 1.15–2.66), and 1.62 (95% CI = 1.06–2.47), respectively.

## 4. Discussion

To the best of our knowledge, the current study was the first to examine the association between perceived neighborhood environment and sarcopenia in urban-dwelling older adults, which found that five environmental factors (no access to public transport, poor recreational facilities, absence of destination, many hill hazards, and lack of traffic safety) were significantly associated with sarcopenia.

We confirmed the relationship between physical frailty and perceived neighborhood environment in our previous study [12]. Sarcopenia is a core component of physical frailty [36] and is a common risk factor [5]. Therefore, perceived neighborhood environment that reduces the risk of physical frailty is likely to be related to sarcopenia. Our study found that after adjusting for potential confounding variables, a low IPAQ-E total score was associated with an increased prevalence of sarcopenia. Okuyama et al. confirmed whether neighborhood environmental factors, such as hilliness, bus stop density, intersection density, residential density, and distance to community centers, were associated with muscle mass and grip strength in older adults after a 3-year follow-up period [18]. In the Okuyama’s study, the measurement of the neighborhood environment was calculated within a 1000-m network buffer from each participant’s residence location based on an actual street network using geographic information systems [18]. However, our study differed from previous studies in that it was evaluated with comprehensive and perceived neighborhood environment tools including aesthetic environment, safety, infrastructure (sidewalks, bike lanes) and social environment. In addition, a systematic review identified a positive association between the neighborhood-built environment and physical function, although physical function was measured using basic ADL [37]. These studies were evaluated sarcopenia components or self-reported physical ability, and not sarcopenia itself. Therefore, our study confirmed that the association differs from previous studies of sarcopenia itself, including the perceived neighborhood environment total score and the individual components. Our results showed that perceived neighborhood environment—especially factors of no access to public transport, poor recreational facilities, absence of destination, many hill hazards, and lack of traffic safety—were related to sarcopenia, even after controlling for potential covariates.

Our study found that poor access to public transport was the most significant neighborhood environmental factor contributing to sarcopenia (46.0% [45.2−46.8]). Access to public transport means that it is less than a 10–15-min walk to a transit station (bus stop, train subway station) from a participant’s home. In a previous study, the use of public transportation increased the amount of walking activity, which decreased the risk of obesity and reduced sedentary time [38]. Thus, the use of public transportation can promote an active lifestyle for older adults. Previous studies have shown that public transportation is essential for older people with poor health (for example, functional impairment) because it creates an environment that is easy to navigate on their own, without depending on others [39]. In addition, our results indicated that physical environmental factors, including hill hazards, traffic safety, and recreational facilities, might be associated with sarcopenia. Negative perceptions of these environments may lead to lower physical activity due to restrictions in using active life spaces [39,40]. Several studies have shown that physical inactivity may be associated with an increased risk of chronic diseases, modifications in body composition (increased fat and decreased muscle mass), and premature death [41,42,43]. A systematic review also showed that inactive older persons had an increased risk of sarcopenia [44]. Therefore, the poorer the perception of one’s physical environment, the higher the risk of sarcopenia due to lower physical activity levels.

Our study also showed that the absence of a destination was significantly associated with sarcopenia. The absence of a destination referred to the lack of public facilities, such as banks and post offices, that provide opportunities for social activities. Previous studies have shown that a lack of social resources can induce physical and social disabilities due to limited social integration and cohesion [45]. Therefore, the presence of a destination may allow older people to participate in social activities, which in turn may contribute to a healthy life. Specifically, one study showed that social support positively affected muscle mass, muscle strength, and physical function, which are components of sarcopenia [46]. In addition, the presence of a destination includes access to medical services. Thus, people who perceive insufficient access to medical services may experience difficulties in healthcare access [47]. For example, due to the lack of information on health, preventive care as well as opportunities for health maintenance and early treatment can be neglected [48,49]. Therefore, an environment that has no social attributes, such as absence to destinations, can affect sarcopenia in two ways: (i) lowering social interactions and (ii) interrupting one’s ability to control their health.

This study had several limitations. First, our study participants were older adults that were recruited from an ambulatory community and were relatively healthy. Thus, our findings may not be applicable to other settings and populations. Second, our information on the perceived neighborhood environment was obtained by self-reported questionnaires using the IPAQ-E. We measured the perceived environment factors which might be independent of the objective environment characteristics. The objective measures are less biased and less fluctuating aspects of the environment, whereas perceived measures of the environment are influenced by social and cognitive factors [50]. Nevertheless, both objective and perceived neighborhood environmental attributes have been related to physical activity and behavior [51]. In our study, we found that poor perceived neighborhood environments were associated with sarcopenia, even after controlling for psychosocial and social factors as confounding variables. Furthermore, the IPAQ-E questionnaire has been suggested as reliable assessment tool [24], which has been used in several countries [21,22,23]. Moreover, perceived neighborhood environment may be shown to predict health and health behavior more than the objectively measured environment among older adults, making it convenient to evaluate various neighborhood environmental variables [50]. Future research is needed to investigate the relationship between direct and indirect neighborhood environment to sarcopenia through objective and perceived evaluation of the neighborhood environment.

Third, the cross-sectional study design did not allow us to establish causal relationships between the perceived neighboring environment and two variables of sarcopenia. In the future, a longitudinal study design is needed to substantiate this relationship. Nevertheless, this study had major strengths, such as including a large nationally representative sample of community-dwelling older Korean adults, using a comprehensive measure of the perceived neighborhood environment (analysis of environmental factors considering the characteristics of the neighborhood environment), and including various covariates in the analysis.

## 5. Conclusions

Our study findings indicate that poor perceived neighborhood environments were associated with sarcopenia among urban-dwelling older adults, even after controlling for physical health conditions as confounding variables. Furthermore, perceived neighborhood environment factors, including physical activity and social participation, were related to sarcopenia. Our findings have implications in the prevention and management of sarcopenia among older adults for city planners and public health workers as they add to the literature on the association between the neighborhood-built environment and sarcopenia.

## Figures and Tables

**Table 1:** Characteristics of participants in the non-sarcopenia and sarcopenia groups.

Variables	Overall (*n* = 1778)			Non-Sarcopenia Group (*n* = 1378)			Sarcopenia Group (*n* = 400)			*p*-Value ^†^	*OR*	(95% CI) ^‡^
**Sociodemographic factors**												
Age (years)	75.9	±	3.8	75.5	±	3.7	77.2	±	3.8	<0.001	1.12	(1.09−1.16) ***
70−74	714	(40.2)		612	(44.4)		102	(25.5)		<0.001	1.00	
75−79	667	(37.5)		508	(36.9)		159	(39.8)			1.88	(1.43−2.47) ***
≥80	397	(22.3)		258	(18.7)		139	(34.8)			3.23	(2.41−4.34) ***
Female sex (%)	961	(54.0)		769	(55.8)		192	(48.0)		0.006	0.73	(0.58−0.91) *
Education (years)	9.6	±	4.9	9.6	±	4.9	9.8	±	4.9	0.381	1.01	(0.99−1.03)
Living alone	376	(21.1)		296	(21.5)		80	(20.0)		0.523	0.91	(0.69−1.21)
Marital status (without partner)	562	(31.6)		445	(32.3)		117	(29.3)		0.249	0.87	(0.68−1.11)
Socioeconomic status	136	(7.6)		110	(8.0)		26	(6.5)		0.326	0.80	(0.51−1.25)
**Health-related factors**												
Current smoker	89	(5.0)		57	(4.1)		32	(8.0)		0.002	2.02	(1.29−3.15) **
Alcohol consumption (≥2–3 times/week)	312	(17.5)		234	(17.0)		78	(19.5)		0.244	1.18	(0.89−1.57)
Low physical activity	154	(8.7)		101	(7.3)		53	(13.3)		<0.001	1.93	(1.36−2.75) ***
Poor nutritional status (MNA-SF score of ≤11)	134	(7.5)		84	(6.1)		50	(12.5)		<0.001	2.20	(1.52−3.18) ***
Hypertension	1047	(58.9)		806	(58.5)		241	(60.3)		0.529	1.08	(0.86−1.35)
Diabetes	406	(22.8)		295	(21.4)		111	(27.8)		0.008	1.41	(1.09−1.82) *
Cardiovascular diseases	223	(12.5)		176	(12.8)		47	(11.8)		0.587	0.91	(0.65−1.28)
Osteoporosis	295	(16.6)		215	(15.6)		80	(20.0)		0.037	1.35	(1.02−1.80) *
Number of comorbidities	1.8	±	1.3	1.8	±	1.3	1.9	±	1.3	0.147	1.07	(0.98−1.16)
Number of medications	3.5	±	2.8	3.4	±	2.8	3.9	±	2.8	<0.001	1.07	(1.03−1.11) ***
BMI (kg/m^2^)	24.5	±	2.9	25.0	±	2.9	23.0	±	2.7	<0.001	0.77	(0.74−0.81) ***
<18.5	34	(1.9)		14	(1.0)		20	(5.0)		<0.001	1.00	
18.5−24.9	1021	(57.4)		724	(52.5)		297	(74.3)			0.29	(1.14−0.58) ***
≥25.0	723	(40.7)		640	(46.4)		83	(20.8)			0.09	(0.04−0.19) ***
**Psychosocial factors**												
Depressive symptoms (SGDS-K score of ≥6)	378	(21.3)		265	(19.2)		113	(28.2)		<0.001	1.65	(1.28−2.14) ***
Fair/poor self-perceived health	472	(26.5)		327	(23.7)		145	(36.3)		<0.001	1.83	(1.44−2.32) ***
Cognitive function (MMSE score of <24)	288	(16.2)		201	(14.6)		87	(21.8)		0.001	1.63	(1.23−2.16) ***
**Social factors**												
Participation in social meetings (yes)	1661	(93.4)		1296	(94.0)		365	(91.3)		0.047	1.52	(1.00−2.29) *
**Sarcopenia**												
Handgrip strength (kg)	26.5	±	7.4	27.2	±	7.6	24.2	±	6.4	<0.001	0.94	(0.93−0.96) ***
Usual gait speed (m/s)	1.1	±	0.2	1.2	±	0.3	1.0	±	0.2	<0.001	0.08	(0.04−0.13) ***
Five-times sit-to-stand score (s)	11.1	±	3.7	10.5	±	3.4	13.0	±	3.8	<0.001	1.19	(1.15−1.23) ***
SPPB score	11.0	±	1.3	11.2	±	1.3	10.4	±	1.4	<0.001	0.68	(0.62−0.73) ***
SMI—appendicular (kg/m^2^)	6.4	±	1.0	6.6	±	1.0	5.7	±	0.8	<0.001	0.30	(0.25−0.35) ***
**Neighborhood environment**												
IPAQ-E total score (16−65)	55.1	±	7.3	55.3	±	7.2	54.2	±	7.6	0.005	0.98	(0.96−0.98) **
Residential density (0−1)	0.7	±	0.5	0.7	±	0.5	0.7	±	0.5	0.346	0.89	(0.70−1.13)
Access to destinations (5−20)	17.7	±	2.5	17.8	±	2.4	17.2	±	2.8	<0.001	0.91	(0.87−0.95) ***
Neighborhood infrastructure (4−16)	13.5	±	2.7	13.5	±	2.7	13.5	±	2.6	0.925	1.00	(0.96−1.04)
Neighborhood safety (4−16)	13.3	±	2.5	13.4	±	2.5	13.1	±	2.6	0.035	0.95	(0.91−1.00) *
Social environment (1−4)	3.5	±	0.8	3.5	±	0.8	3.4	±	0.8	0.246	0.93	(0.81−1.05)
Aesthetic qualities (1−4)	3.1	±	1.1	3.1	±	1.1	3.0	±	1.1	0.118	0.92	(0.84−1.02)
Street connectivity (1−4)	3.3	±	0.9	3.3	±	0.9	3.3	±	0.9	0.838	0.99	(0.87−1.12)

Values are presented as either means ± standard deviations or *n* (%). IPAQ-E = International Physical Activity Questionnaire Environment Module; BMI = body mass index; IADL = Instrumental Activities Daily Living; SGDS-K = Korean Version of Short Form Geriatric Depression Scale; MMSE = Mini-Mental State Examination; MNA-SF = Mini Nutritional Assessment Short Form; SPPB = Short Physical Performance Battery; SMI = Skeletal muscle mass index; CI = confidence interval; OR = odds ratio. ^†^ *p*-values are based on the chi-square test or independent t-test. * *p* < 0.05, ** *p* < 0.01, *** *p* < 0.005. ^‡^ Association between characteristics of participants by non-sarcopenia and sarcopenia groups by univariate logistic regression analysis.

**Table 2:** Adjusted odds ratios of neighborhood environmental factors for sarcopenia (*n* = 1778).

Variables	Category	Total Sample			Model 1			Model 2			Model 3			Model 4	
		*n*	(%)	*OR*	(95% CI)	*p*	*OR*	(95% CI)	*p*	*OR*	(95% CI)	*p*	*OR*	(95% CI)	*p*
**Residential density**															
Residential density	High	1256	(70.6)	1.00		0.562	1.00		0.442	1.00		0.535	1.00		0.534
	Low	522	(29.4)	1.08	(0.83−1.39)		1.11	(0.85−1.45)		1.09	(0.83−1.43)		1.09	(0.83−1.43)	
**Access to destinations**															
Access to shops	Good	1654	(93.0)	1.00		0.715	1.00		0.813	1.00		0.989	1.00		0.999
	Poor	124	(7.0)	1.09	(0.70−1.68)		1.06	(0.67−1.67)		1.00	(0.63−1.59)		1.00	(0.63−1.59)	
Access to public transport	Yes	1706	(96.0)	1.00		0.001	1.00		0.003	1.00		0.009	1.00		0.009
	No	72	(4.0)	2.36	(1.42−3.91)		2.25	(1.33−3.81)		2.04	(1.19−3.48)		2.04	(1.19−3.48)	
Access to recreational facilities	Good	1484	(83.5)	1.00		0.004	1.00		0.015	1.00		0.038	1.00		0.042
	Poor	294	(16.5)	1.55	(1.15−2.09)		1.47	(1.08−2.01)		1.40	(1.02−1.91)		1.39	(1.01−1.90)	
Presence of destination	Yes	1590	(89.4)	1.00		0.001	1.00		0.005	1.00		0.022	1.00		0.022
	No	188	(10.6)	1.79	(1.28−2.52)		1.66	(1.16−2.37)		1.53	(1.06−2.20)		1.53	(1.06−2.20)	
Hill hazards	No	1316	(74.0)	1.00		0.108	1.00		0.029	1.00		0.030	1.00		0.031
	Yes	462	(26.0)	1.24	(0.95−1.61)		1.36	(1.03−1.78)		1.36	(1.03−1.79)		1.36	(1.03−1.78)	
**Neighborhood infrastructure**															
Presence of sidewalks	Yes	1709	(96.1)	1.00		0.189	1.00		0.341	1.00		0.381	1.00		0.380
	No	69	(3.9)	1.46	(0.83−2.55)		1.32	(0.74−2.37)		1.30	(0.72−2.33)		1.30	(0.72−2.34)	
Presence of bike lanes	Yes	1152	(64.8)	1.00		0.530	1.00		0.509	1.00		0.502	1.00		0.542
	No	626	(35.2)	1.08	(0.84−1.39)		1.09	(0.84−1.41)		1.09	(0.84−1.42)		1.09	(0.83−1.41)	
Maintenance of sidewalks	Good	1643	(92.4)	1.00		0.824	1.00		0.963	1.00		0.934	1.00		0.945
	Poor	135	(7.6)	1.05	(0.68−1.64)		0.99	(0.62−1.57)		0.98	(0.62−1.56)		0.98	(0.62−1.57)	
Maintenance of bike lanes	Good	1291	(72.6)	1.00		0.197	1.00		0.241	1.00		0.193	1.00		0.189
	Poor	487	(27.4)	0.84	(0.64−1.10)		0.84	(0.64−1.12)		0.83	(0.62−1.10)		0.83	(0.62−1.10)	
**Neighborhood safety**															
Crime safety at night	Safe	1451	(81.6)	1.00		0.665	1.00		0.583	1.00		0.698	1.00		0.715
	Not safe	327	(18.4)	1.07	(0.79−1.44)		1.09	(0.80−1.49)		1.06	(0.78−1.46)		1.06	(0.77−1.45)	
Traffic safety	Safe	1356	(76.3)	1.00		0.067	1.00		0.017	1.00		0.040	1.00		0.038
	Not safe	442	(23.7)	1.28	(0.98−1.67)		1.40	(1.06−1.85)		1.34	(1.01−1.78)		1.35	(1.02−1.78)	
Traffic safety for bicyclists	Safe	897	(50.4)	1.00		0.210	1.00		0.097	1.00		0.171	1.00		0.175
	Not safe	881	(49.6)	1.16	(0.92−1.46)		1.23	(0.96−1.56)		1.19	(0.93−1.52)		1.19	(0.93−1.52)	
Crime safety during the day	Safe	1690	(95.1)	1.00		0.673	1.00		0.854	1.00		0.964	1.00		0.960
	Not safe	88	(4.9)	1.12	(0.66−1.89)		1.05	(0.61−1.80)		1.01	(0.59−1.73)		1.01	(0.59−1.73)	
**Social environment**															
Seeing people being active	Yes	1543	(86.8)	1.00		0.759	1.00		0.624	1.00		0.469	1.00		0.441
	No	235	(13.2)	0.95	(0.67−1.33)		0.92	(0.64−1.31)		0.88	(0.61−1.25)		0.87	(0.61−1.24)	
**Aesthetic qualities**															
Aesthetics	Yes	1334	(75.0)	1.00		0.075	1.00		0.094	1.00		0.255	1.00		0.278
	No	444	(25.0)	1.27	(0.98−1.66)		1.27	(0.96−1.67)		1.18	(0.89−1.56)		1.17	(0.88−1.55)	
**Street connectivity**															
Connectivity of streets	Yes	1499	(84.3)	1.00		0.902	1.00		0.707	1.00		0.722	1.00		0.685
	No	279	(15.7)	0.98	(0.71−1.35)		0.94	(0.67−1.31)		0.94	(0.67−1.32)		0.93	(0.66−1.31)	

OR = odds ratio; CI = confidence interval. Model 1: Adjusted for age, sex, education years, living alone, marital status (without partner), social security recipient, and DXA equipment used (Whole body DXA Hologic and Whole body DXA Lunar). Model 2: Further adjusted for smoking status, alcohol consumption, number of comorbidities (self-reported doctor diagnosis of hypertension, myocardial infarction, dyslipidemia, diabetes mellitus, congestive heart failure, angina pectoris, peripheral vascular disease, cerebrovascular disease, osteoarthritis, rheumatoid arthritis, osteoporosis, asthma, and chronic obstructive pulmonary disease), nutritional status (Mini Nutritional Assessment Short Form score), low physical activity, and body mass index (BMI). Model 3: Further adjusted for depressive symptoms and cognitive function (Mini-Mental State Examination total score). Model 4: Further adjusted for participation in social meetings (social capital).

## Data Availability

Data can be provided on request from corresponding authors.

## Appendix A

**Table A1:** The 17 items of the International Physical Activity Questionnaire Environmental Module and scoring methods.

Scale Composition	Contents	Response Categories/Scoring Method			
**Residential density (1 item)**		Single-family housing	Apartments with 2–3 stories	Mix of single-family housing and apartments with 2–3 stories	Condos with 4–12 stories, and condos with >13 stories
Type of housing	What is the main type of housing in your neighborhood?	0	1	1	1
**Access to destinations (5 items)**		Strongly disagree	Somewhat disagree	Somewhat agree	Strongly agree
Access to shops	Many shops are within walking distance of my home	1	2	3	4
Access to public transport	It is less than a 10−15-min walk to a transit station from my home	1	2	3	4
Access to recreational facilities	My neighborhood has several free or low-cost recreational facilities	1	2	3	4
Presence of destinations	There are many destinations around the house, such as banks, post offices, medical institutions, and public facilities	1	2	3	4
Hill hazards *	It is not easy to walk to your destination because of the many hills or slopes around your house	4	3	2	1
**Neighborhood infrastructure (4 items)**		Strongly disagree	Somewhat disagree	Somewhat agree	Strongly agree
Presence of sidewalks	There are sidewalks on most of the streets in my neighborhood	1	2	3	4
Presence of bike lanes	There are facilities to cycle in or near my neighborhood	1	2	3	4
Maintenance of sidewalks	The sidewalks in my neighborhood are well-maintained and not obstructed	1	2	3	4
Maintenance of bike lanes	Places for cycling in and around my neighborhood are well-maintained and not obstructed	1	2	3	4
**Neighborhood safety** **(4 items)**		Strongly disagree	Somewhat disagree	Somewhat agree	Strongly agree
Crime safety at night *	The crime rate in my neighborhood makes it unsafe to go on walks at night	4	3	2	1
Traffic safety *	There is so much traffic on the streets that walking is difficult or unpleasant	4	3	2	1
Traffic safety for bicyclists *	There is so much traffic on the streets that it makes it difficult or unpleasant to ride a bicycle in my neighborhood	4	3	2	1
Crime safety during the day *	The crime rate in my neighborhood makes it unsafe to go on walks during the day	4	3	2	1
**Social environment (1 item)**		Strongly disagree	Somewhat disagree	Somewhat agree	Strongly agree
Seeing people being active	I see many people being physically active in my neighborhood	1	2	3	4
**Aesthetic qualities (1 item)**		Strongly disagree	Somewhat disagree	Somewhat agree	Strongly agree
Aesthetics	There are many interesting things to look at while walking in my neighborhood	1	2	3	4
**Street connectivity (1 item)**		Strongly disagree	Somewhat disagree	Somewhat agree	Strongly agree
Connectivity of streets	There are many four-way intersections in my neighborhood	1	2	3	4
Total possible score = 65					

Scores were dichotomized as follows: 1−2 points = 0 point; 3−4 points = 1 point. * Reverse scoring.

**Table A2:** Attributable risk (AR) and population attributable risk (PAR) of neighborhood environmental five factors for sarcopenia (*n* = 1778).

Variables	Sarcopenia (*n* = 400)	Non-Sarcopenia (*n* = 1378)	RR for Sarcopenia (95% CI)	Attributable Risk (AR%)	Population AR (PAR%)
**Access to public transport**					
Yes (ref)	371	1335	1.00		
No	29	43	1.85 (1.83–1.87)	46.0% (45.2–46.8)	3.3%
**Access to recreational facilities**					
Good (ref)	317	1167	1.00		
Poor	83	211	1.32 (1.31–1.33)	24.3% (23.7–24.9)	5.0%
**Presence of destination**					
Yes (ref)	339	1251	1.00		
No	61	127	1.52 (1.51–1.53)	34.3% (33.7–34.9)	5.2%
**Hill hazards**					
No (ref)	279	1037	1.00		
Yes	121	341	1.24 (1.23–1.24)	19.1% (18.5–19.6)	5.8%
**Traffic safety**					
Safe (ref)	291	1065	1.00		
Not safe	109	313	1.20 (1.20–1.21)	16.9% (16.4–17.5)	4.6%

RR = relative risk; AR = attributable risk; PAR = population attributable risk.
